# Regulation of Chemokine Activity – A Focus on the Role of Dipeptidyl Peptidase IV/CD26

**DOI:** 10.3389/fimmu.2016.00483

**Published:** 2016-11-11

**Authors:** Mieke Metzemaekers, Jo Van Damme, Anneleen Mortier, Paul Proost

**Affiliations:** ^1^Laboratory of Molecular Immunology, Department of Microbiology and Immunology, Rega Institute, KU Leuven, Leuven, Belgium

**Keywords:** chemokine, leukocyte migration, GPCR, glycosaminoglycan, posttranslational modification, proteolysis, dipeptidyl peptidase IV, CD26

## Abstract

Chemokines are small, chemotactic proteins that play a crucial role in leukocyte migration and are, therefore, essential for proper functioning of the immune system. Chemokines exert their chemotactic effect by activation of chemokine receptors, which are G protein-coupled receptors (GPCRs), and interaction with glycosaminoglycans (GAGs). Furthermore, the exact chemokine function is modulated at the level of posttranslational modifications. Among the different types of posttranslational modifications that were found to occur *in vitro* and *in vivo*, i.e., proteolysis, citrullination, glycosylation, and nitration, NH_2_-terminal proteolysis of chemokines has been described most intensively. Since the NH_2_-terminal chemokine domain mediates receptor interaction, NH_2_-terminal modification by limited proteolysis or amino acid side chain modification can drastically affect their biological activity. An enzyme that has been shown to provoke NH_2_-terminal proteolysis of various chemokines is dipeptidyl peptidase IV or CD26. This multifunctional protein is a serine protease that preferably cleaves dipeptides from the NH_2_-terminal region of peptides and proteins with a proline or alanine residue in the penultimate position. Various chemokines possess such a proline or alanine residue, and CD26-truncated forms of these chemokines have been identified in cell culture supernatant as well as in body fluids. The effects of CD26-mediated proteolysis in the context of chemokines turned out to be highly complex. Depending on the chemokine ligand, loss of these two NH_2_-terminal amino acids can result in either an increased or a decreased biological activity, enhanced receptor specificity, inactivation of the chemokine ligand, or generation of receptor antagonists. Since chemokines direct leukocyte migration in homeostatic as well as pathophysiologic conditions, CD26-mediated proteolytic processing of these chemotactic proteins may have significant consequences for appropriate functioning of the immune system. After introducing the chemokine family together with the GPCRs and GAGs, as main interaction partners of chemokines, and discussing the different forms of posttranslational modifications, this review will focus on the intriguing relationship of chemokines with the serine protease CD26.

## Introduction

*Mammalia* are exposed to a variety of pathological agents on a daily basis and disease-causing microorganisms such as bacteria and viruses are omnipresent. Furthermore, cells of the own body can acquire malignant potencies, which make them dangerous with respect to the normal physiology. Since the body is equipped with a protective system, confrontation with pathological stimuli not necessary results in disease. The immune system holds a non-specific, innate component that constitutively monitors one’s health (1, 2). The process of phagocytosis plays a central role in this innate immune system. Phagocytosis by non-specific cells such as macrophages induces the clearance of bacteria (3). Meanwhile, virally infected cells are attacked by natural killer (NK) cells and viral spread is inhibited by interferons (IFNs) (4). The second component of the immune system is adaptive and requires prior activation and B- and T cell proliferation (5–9). As a consequence, the adaptive immune system generates only a slow response upon contact with a particular microorganism for the first time. However, adaptive immunity is characterized by memory, which enables fast induction of a highly specific response when the organism is exposed to the same pathogen in the future (5, 6). In general, the adaptive immune system is subdivided into humoral and cellular immunity, with B and T lymphocytes being the most important effector cells, respectively. Helper T lymphocytes stimulate B lymphocytes to produce antibodies against epitopes that are foreign to the body. The capacity to produce antibodies makes the B lymphocytes important players in immune defense against extracellular pathogens. In addition, cytotoxic and helper T lymphocytes are responsible for combatting intracellular microorganisms (7, 8). Furthermore, regulatory T lymphocytes are crucial for maintaining tolerance to commensal microflora (9).

Leukocytes are crucial for correct functioning of host protection. Different leukocytes, i.e., neutrophils, eosinophils, basophils, lymphocytes, NK cells, monocytes, macrophages, and dendritic cells, have subtype-specific shapes and functions. Obviously, the presence of the correct cells on the right place at the right time is essential to allow the desired interactions between the different leukocyte subtypes and between leukocytes and pathogens resulting in proper functioning of the immune system (10–12). On the one hand, hyperactivation of the immune system can result in allergic or autoimmune responses. On the other hand, immune incompetence has been associated with a significantly increased risk of developing disease. Moreover, inadequate immunity significantly reduces the natural antitumor response. In order to avoid detrimental consequences that result from inappropriate immunological responses, directional migration of leukocytes in healthy individuals is a dynamic highly controlled process that is regulated by adhesion molecules and chemotactic cytokines or chemokines. Chemokines drive migration in a concentration- and site-dependent manner (13–18). The function and biological availability of chemokines and their receptors is modulated at multiple levels including transcription and translation (13). Concerning the dynamic process of chemokine regulation, it became more and more clear that also posttranslational modifications play an important role (19).

## Chemokines

### Definition and Classification of Chemokines

Chemokines are small, chemotactic molecules with a molecular weight of about 7–12 kDa. They direct migration of leukocytes during inflammation as well as in homeostatic circumstances (13, 14, 16, 17). Rolling of leukocytes is followed by lose adhesion of the cells to the endothelium (10). Selectins play an important role in generating primary adhesion interactions. Next, interaction of leukocytes with chemokines strengthens bonding between integrins on leukocytes and their counter-receptors on endothelial cells, resulting in anchorage of leukocytes to the endothelium. In the end, a chemotactic gradient will act as a guide that navigates leukocytes to their final destinations (10, 20, 21).

Although, the mutual sequence homology of chemokines varies between less than 20% to over 90%, the tertiary structure of chemokines, in general, is quite similar (15–17). Most chemokines contain four cysteine residues that form disulfide bridges, which stabilize the tertiary structure of the protein. Chemokines contain a characteristic flexible NH_2_-terminal region of about 6–10 amino acids that is important for signal transduction. An NH_2_ terminal loop (N loop), that mouths into a 3_10_-helix, is situated behind the flexible NH_2_-terminal region. The NH_2_-terminal residues and N loop contain determinants for binding of the chemokine to G protein-coupled receptors (GPCRs) and are followed by a three stranded β-sheet and a COOH-terminal helix (15). Classically, one distinguishes CXC, CC, C, and CX_3_C chemokines. The division into four subfamilies is based on the difference in localization of the two NH_2_-terminal cysteine residues and, consequently, is based on structural characteristics (13, 16, 18).

Most CXC or α-chemokines are clustered on chromosome 4q12–21 and contain only one random amino acid (X) between the two NH_2_-terminal cysteines (16). The human CXC subfamily consists of 18 genes encoding 18 proteins (CXCL1 to 17 and CXCL4L1) that are further subdivided based on the presence or absence of a Glu-Leu-Arg (ELR) motif located just before the CXC motif. ELR^+^CXC chemokines, i.e., CXCL1 to CXCL3 and CXCL5 to CXCL8, interact with CXC chemokine receptor (CXCR)1 and/or CXCR2, thereby mediating migration and activation of neutrophils. In general, members of this group of CXC chemokines also promote angiogenesis. This angiogenic activity partially explains why the pro-inflammatory CXCL8, or interleukin (IL)-8, has been associated with cancer (22, 23). Moreover, it has been demonstrated that CXCL8 stimulates migration of colorectal tumor cells *in vitro* as well as *in vivo* (24). Absence of the ELR motif, in contrast, implies completely different characteristics. The ELR negative (ELR^−^) CXC chemokines CXCL9 to CXCL11, for example, activate the receptor CXCR3 and mediate recruitment of T lymphocytes and NK cells (13). In contrast to the ELR^+^ relatives, the CXCR3 ligands have angiostatic properties. The first described chemokine ever, namely platelet factor-4 or CXCL4, is an ELR^−^CXC chemokine (25). CXCL4, released by activated blood platelets, is a weak CXCR3 ligand and chemoattractant for neutrophils, monocytes, and fibroblasts (26, 27). Due to its extremely high affinity for heparin, CXCL4 acts as a neutralizing agent for heparin-like molecules and hinders the thrombin inactivating capacity of these agents (28, 29). Moreover, it had been shown that CXCL4 inhibits endothelial cell proliferation, angiogenesis, and tumor growth (29–31). The highly related non-allelic variant of platelet factor-4, CXCL4L1, is an even better inhibitor of angiogenesis (32).

The second large subclass of human chemokines contains about 30 CC or β-chemokines. Individual members of this chemokine subfamily all contain an analogous CC motif and most CC chemokines are clustered on chromosome 17, suggesting that the CC family arose as a result of gene duplication (13–16). CC or β-chemokines are subdivided into two main subgroups. One subfamily is the monocyte chemotactic proteins (MCPs) together with the eotaxins (33). These chemokines mediate recruitment of, among others, monocytes, T lymphocytes, eosinophils, and basophils and promote histamine release by the latter. Consequently, they play an important role in inflammation including allergic inflammation (34). Most other CC chemokines are considered as a separate CC chemokine subclass with high homology to the macrophage inflammatory proteins (MIP)-1α and MIP-1β (14).

In contrast to CXC and CC chemokines, C chemokines contain only two cysteines. Indeed, as a result, their overall topology is stabilized by only one disulfide bridge. Nowadays, XCL1 and XCL2, also known as lymphotactin α and β, respectively, are the only C chemokines that have been identified (13–17). CX_3_CL1 – or fractalkine – is still the only CX_3_C chemokine that has been described. This chemokine not only acts as a chemoattractant but also plays a role as an adhesion molecule (35). Structurally, CX_3_CL1 contains an NH_2_-terminal chemokine domain, a long mucin-like domain of circa 110 amino acids that is enriched for serine and threonine residues, a transmembrane domain, and a cytoplasmic tail. CX_3_CL1 is capable of anchoring to extracellular surfaces and is – together with the membrane-bound CXCL16 – an exception to the rule that chemokines are secreted proteins (15).

In addition to the structure-based classification of chemokines as CXC, CC, C, and CX_3_C family members, one respects a functional division between inflammatory and homeostatic chemokines (13, 15). Expression of inflammatory chemokines requires prior activation by stimuli that can be exogenous as well as endogenous. Examples of endogenous mediators that are potent inducers of expression of inflammatory chemokines are cytokines, for example, IFN-γ, tumor necrosis factor (TNF)-α, IL-1, IL-4, IL-5, IL-6, IL-13, and IL-17 (14, 15, 17, 18, 36, 37). Well known exogenous stimuli are microbial infection and viral and bacterial components such as the toll-like receptor ligands double stranded RNA, peptidoglycan, and lipopolysaccharide (14, 15, 18, 38, 39). The presence of pathogen-associated molecular patterns (PAMPs) on the surface of these factors facilitates recognition by the body, allowing generation of an appropriate immune response (1, 2). In fact, PAMPs on the surfaces of the infection-associated molecules interact with pattern recognizing receptors, enabling induction of chemokine production and subsequent leukocyte attraction (40). Examples of inflammatory CC chemokines are CCL2 and CCL5 (41).

Homeostatic chemokines control basal cell migration and are constitutively expressed. They enable correct hematopoiesis in bone marrow and thymus and direct migration of lymphocytes and dendritic cells to secondary lymphoid organs (15, 42, 43). Furthermore, homeostatic chemokines are responsible for leukocyte migration in healthy peripheral tissues resulting in immune surveillance and maintenance of mucosal immunity (42–44). Genetic deficiency of CXCL13, for example, results in aberrances in the organization of Peyer’s patches in the intestine (44). In addition to the fact that homeostatic chemokines are necessary for correct functioning of the immune system, they do also play a role in various developmental processes (15, 42, 43). Migration through specific areas in secondary lymphoid organs during B and T lymphocyte development, for example, is navigated by homeostatic chemokines (42). Noteworthy, it is recommended to interpret the broad, function-based chemokine subdivision with some caution. The distinction between homeostatic and inflammatory chemokines is not absolute: some chemokines can fall into both categories, depending on the context (15, 45). On top of their role as leukocyte attractants, a number of chemokines (homeostatic and inflammatory) have also been reported to have direct chemokine receptor-independent antimicrobial activity [reviewed in Ref. (46)]. The direct antimicrobial activity in general requires higher concentrations compared to the concentrations needed to induce leukocyte migration (47, 48).

### Chemokine Receptors

**Figure 1:**
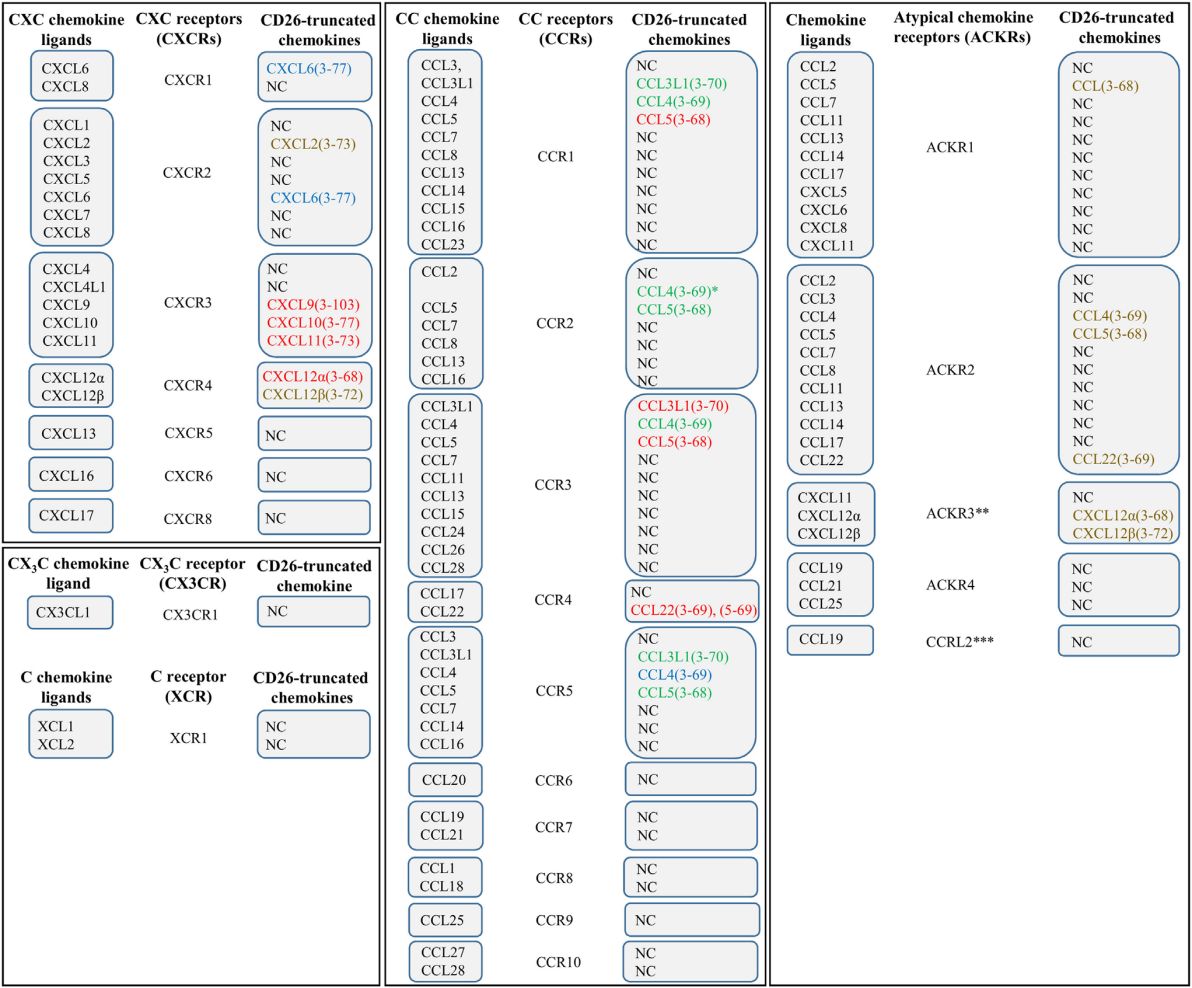
**The chemokine family and effect of CD26 on chemokine receptor–ligand interactions**. Various chemokines can be subjected to proteolytic processing by the enzyme dipeptidyl peptidase IV or CD26. The effects of truncation by CD26 are indicated by colors. Red, CD26-mediated proteolysis negatively affects the interaction between chemokine and chemokine receptor. Green, CD26-mediated proteolysis has a positive effect on the interaction between chemokine and chemokine receptor. Blue, proteolytic processing by CD26 does not influence the interaction between chemokine and chemokine receptor. Brown, the implications of truncation by CD26 remain to be determined. *, in contrast to intact CCL4, CCL4(3–69) shows affinity for CCR1 and CCR2. **, also known as CXCR7. ***, the notation “ACKR5” is reserved for this receptor. NC, not cleaved by CD26.

Chemokines exert their chemotactic activity *via* binding and activation of GPCRs. Since ligands of GPCRs are often non-protein molecules and cytokines signal *via* non-GPCRs, the association between chemotactic cytokines and this type of receptors was not self-evident. The fact that neutrophils are characterized by the presence of binding sites with a high affinity for CXCL8, together with the observation that *Pertussis* toxin is able to block the effects of CXCL8, indicated that chemokine receptors are GPCRs (49, 50). Indeed, *Pertussis* toxin is well known for its inactivating effect on the G_i_ type of G proteins. Nowadays, almost 20 chemokine receptors have been cloned within the over 600 human GPCRs. These GPCRs are known as CXCR1 to CXCR6, CXCR8, CC chemokine receptor (CCR)1 to CCR10, CX_3_C chemokine receptor (CX_3_CR)1, and C chemokine receptor (XCR)1. They specifically interact with one or more chemokines of the corresponding subclass (19, 51–53) (Figure 1). CXCR2, for example, is capable of binding all seven known human ELR^+^CXC chemokines, whereas CXCR4 selectively interacts only with one chemokine, namely CXCL12. In addition, some chemokines activate multiple receptors. For example, CCL3L1 and CCL5 show affinity for CCR1, CCR3, and CCR5 (54–56).

**Figure 2:**
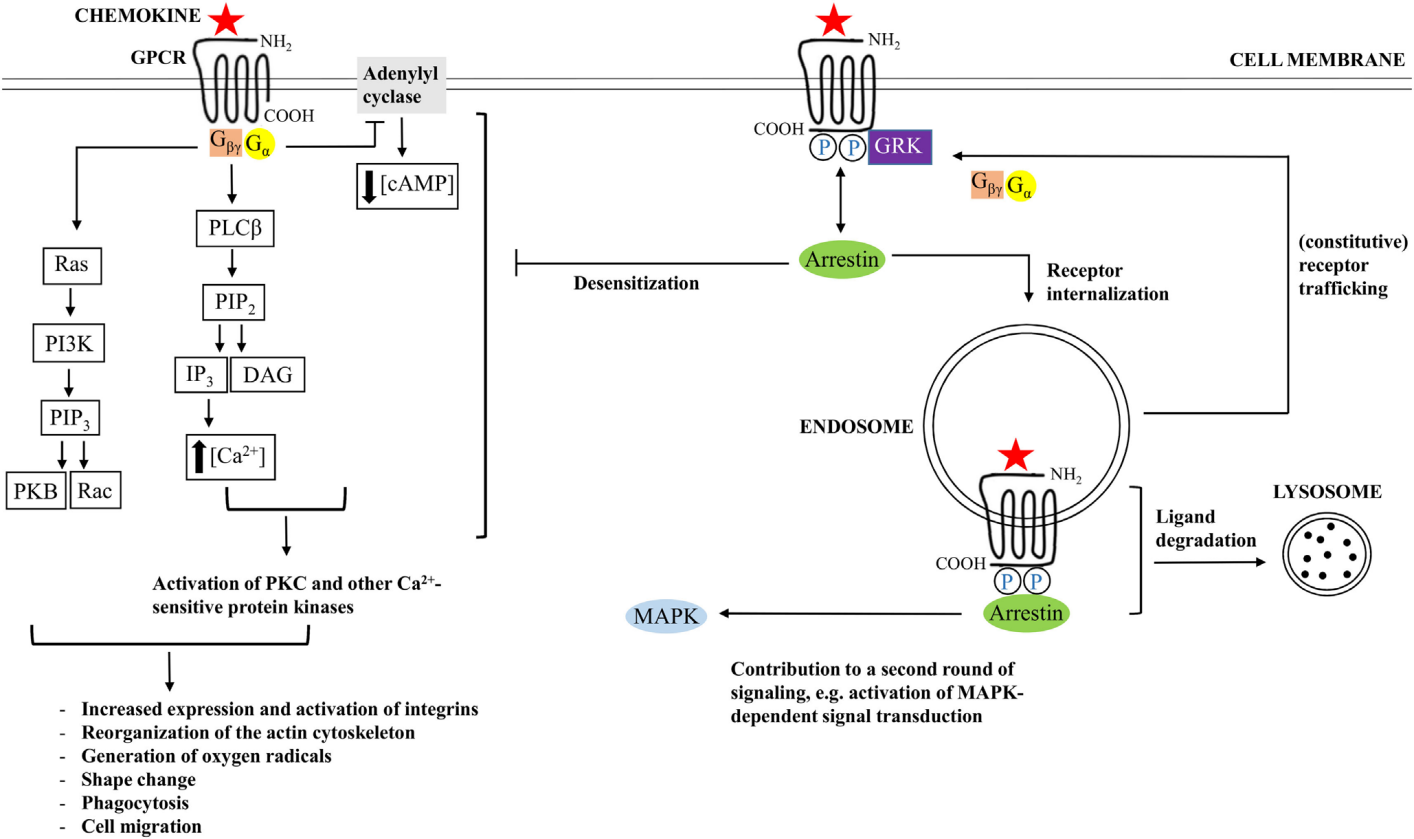
**Chemokine-induced signal transduction**. Chemokine receptors are G protein-coupled receptors (GPCRs), implying that classical chemokine-induced signaling is G protein-dependent. Binding of a chemokine ligand induces a change in conformation of the GPCR, thereby facilitating exchange of guanosine diphosphate (GDP), which is bound by the α-subunit of the G protein during receptor inactivity, for guanosine triphosphate (GTP). Most chemokine receptors are coupled to G proteins that hold an inhibitory type of α subunit (Gα_i_), implying that the newly formed Gα–GTP complex mediates inhibition of adenylyl cyclase, resulting in decreasing cyclic adenosine monophosphate (cAMP) concentrations. The βγ-subunit of the G protein (G_βγ_), in turn, activates phospholipase Cβ (PLCβ), resulting in initiation of cleavage of phosphatidylinositol 4,5-bisphosphate (PIP_2_) into diacylglycerol (DAG) and inositol 1,4,5-trisphosphate (IP_3_). The latter facilitates calcium release from the endoplasmic reticulum. Calcium and PIP_2_ cooperate in the activation of protein kinase C (PKC) and other calcium-sensitive protein kinases. In addition, G_βγ_ interacts with Ras, followed by activation of phosphatidylinositol-3-kinases (PI3K) and PIP_3_. PIP_3_ activates Rac, a GTPase, and interacts with protein kinase B (PKB), which are important for leukocyte migration and actin polymerization, respectively. In the end, modulation of actin-dependent processes regulates various leukocyte functions and initiates chemotaxis. In addition to G protein-dependent signaling, some chemokine receptors couple to arrestin after chemokine-binding and phosphorylation of the receptor by G protein-coupled receptor kinases (GRK). Arrestin mediates G protein uncoupling of the receptor and plays a role in receptor desensitization. In addition, arrestin interaction can promote receptor internalization to endosomes and ligand degradation, initiation of an additional round of cell signaling, or receptor recycling to the cell membrane.

Interaction between a GPCR and a chemokine ligand results in receptor activation, followed by receptor-mediated signal transduction. GPCRs contain seven α-helices that cross the membrane. Individual transmembrane domains are mutually connected by three intracellular and three extracellular loops. The NH_2_-terminal region is located extracellularly, whereas the COOH-terminus faces the cytoplasm (15, 51). The extracellular loops and the NH_2_-terminal residues facilitate activation of a chemokine receptor, and the intracellular loops are crucial for coupling to G proteins (Figure 2). The second intracellular loop contains a so called DRYLAIV-motif that is necessary to enable signal transduction (57, 58).

The G proteins are receptor-associated when the receptor is inactive. As long as the receptor is kept in its inactive conformation, the guanosine triphosphatase (GTPase) domain of the α-subunit of the G protein is bound to guanosine diphosphate (GDP). However, binding of a suitable chemokine ligand gives rise to a conformational change in the receptor, thereby leading to the exchange of GDP for guanosine trisphosphate (GTP) (58–60). The newly formed Gα–GTP complex dissociates from the receptor and from the βγ subunit of the G protein. With regard to α subunits, one respects a classification into Gα_s_, Gα_i_/Gα_o_, Gα_q_/Gα_11_, and Gα_12_/Gα_13_ subtypes. These different subfamilies either stimulate or inactivate adenylylcyclases. Most chemokine receptors are coupled to the Gα_i_ subtype, which implies that binding of a chemokine ligand results in inhibition of adenylylcyclase, followed by a decrease in concentration of the messenger molecule cyclic adenosine monophosphate (cAMP) Figure 2 (60). In addition, βγ subunits activate membrane-bound phospholipase C (PLC)β, which enables induction of cleavage of phosphatidylinositol 4,5-bisphosphate (PIP_2_) into diacylglycerol (DAG) and inositol 1,4,5-trisphosphate (IP_3_). IP_3_, in turn, induces the release of calcium from the endoplasmic reticulum (ER) to the cytoplasm. DAG acts synergistically with calcium and activates various forms of protein kinase C (PKC) and other calcium-sensitive protein kinases. The resulting cascade of protein phosphorylations ultimately leads to the generation of cellular effector responses. In addition to PIP_2_, the related phosphatidylinositol (3–5)-trisphosphate (PIP_3_), also plays an important role in chemokine receptor-induced signal transduction. This particular product of the phosphatidylinositol 3-kinase (PI3K) cascade activates Rac, a small GTPase that is indispensable for leukocyte migration. Furthermore, PIP_3_ contains an interaction site for protein kinase B (PKB). Interaction with this ligand is important for actin polymerization (17). Modulation of actin-dependent, cellular processes, in the end, gives rise to induction of chemotaxis and regulation of a variety of other functions in different types of leukocytes (15, 51).

In addition to classical G protein-dependent signaling, some chemokine receptors can couple to arrestin proteins Figure 2 (61–63). These adaptor proteins may bind the receptor following agonist binding and receptor phosphorylation by G protein-coupled receptor kinases (GRKs). By means of sterically hindering the phosphorylated receptor and its interaction with G proteins, arrestin proteins uncouple the receptor from G proteins and promote receptor desensitization (64). Moreover, arrestin recruitment facilitates targeting of the receptor to clathrin-coated pits that are present at the cell surface (65, 66). As a result, the receptor is internalized to endosomes and the ligand can be subjected to degradation. However, binding of arrestin does not necessarily promote receptor downregulation. Instead, in some cases, this interaction initiates an additional round of signaling. Concerning the CXCL12/CXCR4 axis, for example, β arrestin-2 has been reported to enhance the activation of p38 mitogen-activated protein kinase (MAPK) and extracellular signal-regulated kinase (ERK) that is mediated by CXCR4 (67). In this way, β arrestin-2 plays an important role in CXCR4-mediated chemotaxis. In case a specific chemokine receptor interacts with multiple chemokine agonists, the impact of arrestin binding depends on the particular chemokine that binds to the receptor. For example, internalization of CCR7 following CCL19 binding is arrestin-dependent. However, this does not apply to the CCR7 ligand CCL21 (68). In addition to playing a role in chemokine receptor internalization and desensitization, ligand degradation, and initiation of an additional round of cell signaling, β-arrestin coupling can also mediate receptor recycling, resulting in constitutive trafficking of the receptor from intracellular vesicles to the cellular surface (69–72).

Furthermore, some of the identified chemokine receptors do not have the capacity to initiate classical signal transduction. These atypical chemokine receptors (ACKRs) have been associated with processes of internalization, degradation, neutralization, and transport of chemokines (13, 69). ACKR1 to 4 and CCRL2 do not contain a complete DRYLAIV-motif and seem to play a role in regulation of chemokine availability (13, 73). ACKR1 binds both CC chemokines and CXC chemokines and promotes active transport of inflammatory chemokines from basolateral toward apical surfaces through endothelial cells (74, 75). As a consequence of the interaction with ACKR1, a chemokine ligand is internalized, resulting in stacking of the particular molecule on the apical side. Consequently, chemokine transcytosis that is realized by ACKR1 will result in increased leukocyte migration (75). ACKR1 is not only expressed by a variety of endothelial cells but can also be found on the surface of erythrocytes and epithelial cells (76–78). On erythrocytes, ACKR1 functions as a “sink” receptor (77, 79). This phenomenon facilitates maintenance of low chemokine concentrations in the blood in physiological circumstances, suggesting that the atypical chemokine receptor is important to keep particular chemokines in the circulation (19, 76).

Since the NH_2_-terminal chemokine domain is responsible for binding to GPCRs and activation of these receptors, this extremely flexible region is of high importance for a chemokine’s biological activity. The amino acids in front of the first cysteine residue are important for the receptor selectivity of the chemokine (79, 80). This NH_2_-terminal tail of chemokines interacts with a pocket in the transmembrane part of the receptors. Thus, it is not surprising that minor modification on the chemokine’s NH_2_-terminus may have a profound impact on the activity of these proteins (*vide infra*). In addition, a rigid loop that is located behind the second cysteine residue of the chemokine binds to the NH_2_-terminus of the receptors (81, 82). Accumulating evidence suggests an interplay between both interaction sites, which allows fine tuning of the selection and activation of signaling pathways (80).

For some chemokine receptors, three dimensional (3D) structures are already available. For example, crystal structures of CXCR4 in complex with several receptor antagonists have been mapped (83). Furthermore, the receptor was successfully crystalized in complex with a viral chemokine-like molecule (84). Recently, also improved insights into the 3D structure of CXCR1 were obtained using NMR-spectroscopy (85). Since exact structures of most chemokine receptors are still unknown, one frequently depends on hypothetical models that are based on bovine rhodopsin. This seven transmembrane protein is the prototype class A GPCR, and its 3D structure is completely determined. Class A GPCRs are important drug targets and, consequently, improving insights into the receptor structure facilitates the search for interaction partners with therapeutic applications. Given the central role of chemokines in leukocyte migration, modulation of receptor-ligand interactions in the context of chemokines can be of potential value. Indeed, chemokines are associated with various pathological conditions such as acute and chronic inflammation (including autoimmune diseases) and cancer (41, 82, 86, 87).

Expression of chemokine receptors is strongly regulated and is not necessarily restricted to leukocytes (53). Certain conditions can give rise to ectopic expression on endothelial cells, epithelial cells, neurons, and microglial cells in the brain (88–92). Furthermore, expression of chemokine receptors is highly dynamic, as clearly illustrated by the expression patterns on T lymphocytes and dendritic cells. For T lymphocytes, a direct relation exists between the receptor expression pattern and the fact whether it is a naive or memory cell, whether the cell is activated or in an inactive state, and whether the cell has, e.g., a Th1 or a Th2 identity (93). For dendritic cells, the chemokine receptor expression pattern depends on the maturity of the cell (94). Strict regulation of receptor expression ensures correct positioning of lymphocytes and dendritic cells in peripheral or secondary lymphoid tissues.

### Glycosaminoglycans

In addition to binding to GPCRs, chemokines interact with glycosaminoglycans (GAGs) with low affinity (13, 19). These long, linear, and heterogeneous sulfated polysaccharide chains are strongly negatively charged, making them attractive interaction partners for chemokines, which are generally highly basic. GAGs are present on the cellular surface and in the extracellular matrix. They are usually found as part of a so-called proteoglycan structure in which multiple GAGs are bound to a protein core. Proteoglycans are associated with the cellular membrane and function as a macromolecular cell coating that is known as “glycocalyx.” The exact composition of GAGs is highly variable and depends on the location and cell type, by means of which some degree of selectivity for particular chemokines is ensured (95, 96). The differentiation status and the pathophysiological state seem to be important factors underlying this phenomenon (95, 97). Binding of chemokines to GAGs on the vascular endothelial surface, for example, provokes retention of chemokines, thereby enabling formation of a chemokine gradient, which is important for coordinated leukocyte migration. GAGs are also present at the surface of leukocytes (20). Here, loss of GAGs dramatically decreases the affinity of the cell for chemokines. Indeed, one presumes that the interaction between chemokines and GAGs influences the interaction between the chemokine ligands and their receptors (98). Chemokine binding to GAGs on the surface of leukocytes also has been reported to explain cooperativity between specific chemokines (99). By competition for binding to the same GAG on a leukocyte, one chemokine without a specific GPCR on that particular leukocyte (e.g., CXCL13) may detach the second chemokine (e.g., CCL19) from that GAG and allow this second chemokine to activate its specific GPCR (CCR7 in case of CCL19) with higher efficiency. In addition to these interactions with chemokines, GAGs can bind to a variety of other proteins. Thus, the finding that these heterogeneous macromolecules seem to be associated with many cell biological processes was not surprising (13, 95, 100).

The GAG chain structure is composed of a range of 1 up to 25,000 repeating hexose/hexuronic acid plus hexosamine units. These disaccharide building blocks are subjected to an extensive and variable degree of N- or O-sulfation and/or C5 epimerization. Due to the exact composition, glycoside bond and degree of sulfation and acetylation, a strong variety between different disaccharide units is observed. In general, one discriminates heparan sulfate, heparin, chondroitin sulfate, keratan sulfate, dermatan sulfate, and hyaluronic acid. Excluding heparin and hyaluronic acid, GAGs are generally anchored to the cell membrane by means of proteoglycan structures (13, 95). Among the six different subclasses of GAGs, most of the existing knowledge and research concerns heparan sulfates and heparins. Heparan sulfates are expressed by almost all types of cells, making that heparan sulfate proteoglycans count for about 95% of all proteoglycans (101). The diversity between individual GAGs probably results from the fact that synthesis of GAG polymers is not based on a representative template (100). Instead, the production of GAGs depends on a range of complex enzymatic reactions (100).

The major GAG-binding domain of chemokines is often located in the COOH-terminal region and is physically separated from the receptor activation domain that is situated rather NH_2_-terminally. The observation that these two important interaction domains are localized apart from each other, gave rise to the hypothesis that chemokines can interact with both GAGs and GPCRs at the same time (100, 102). However, the GAG-binding domain is not always restricted to the COOH-terminal region and overlaps partly with the receptor activation domain. Given the acidity that characterizes both chemokine receptors and GAGs, this is not surprising. Arginine, lysine, and – to a lesser extent – also histidine in the COOH-terminal α-helix of chemokines, turned out to be crucial hotspots for GAG binding (103–106). Most of the heparan sulfate-binding chemokines like CCL3, CCL4, and CCL5 have a “BBXB” GAG-binding consensus motif in their 40s loop, where “B” symbolizes an arginine or a lysine residue (100, 103). Both are basic amino acids that facilitate binding of the chemokine to negatively charged GAGs. At the moment, the available knowledge on chemokine-binding sites on GAGs themselves is still limited (20, 100). For CXCL8, for example, it is only known that a minimum GAG length of 18 monosaccharides is crucial. Ideally, a GAG chain contains two N-sulfated regions that both consist of six monosaccharides, which are separated from a region of maximum seven non-sulfated disaccharide subunits (107).

Interaction with GAGs promotes oligomerization of chemokines. Indeed, in addition to monomers that are responsible for receptor activation, chemokines also occur as higher order aggregates (15). The capacity of GAGs to facilitate oligomerization of chemokines was demonstrated *in vitro* for the first time for the chemokines CCL2, CCL3, CCL5, and CXCL8 (20). For example, the CC chemokine CCL2 can be subjected to dimerization, and a dimer variant of the CXC chemokine CXCL8 has also been described (108, 109). Generation of CXCL dimers is based on formation of H-bridges between residues in the first strand of the β-sheets of two individual CXCL subunits (15). Indeed, combination of two CXCL subunits implies that, structurally, the resulting dimer is composed of a six stranded β-sheet. This β-sheet is stabilized by interactions between the COOH-terminal α-helices and the β-sheet of the two individual CXCL subunits. The topology of CCL dimers, in contrast, is remarkably less globular (15). These long and flexible structures arise when residues located in the NH_2_-terminal region of two CCL subunits interact, by means of which a two-stranded antiparallel β-sheet interface is formed (15, 108, 110). The positive effect of GAG binding on oligomerization is in line with the presumption that the negatively charged macromolecules facilitate highly specific and local increases in chemokine concentrations.

Binding of chemokines to GAGs and the resulting oligomerization are important for leukocyte migration. This strictly regulated process is of crucial importance in pathophysiological circumstances as well as from a physiological point of view, and depends on the type of chemotactic molecule, both *in vitro* and *in vivo* (10). *In vitro*, it turned out that the process of chemotaxis not necessarily needs binding of chemokines to GAGs. Mutations in the GAG-binding domain of CCL2, CCL4, and CCL5, for example, do not affect the capacity of these chemokines to mediate generation of a chemotactic gradient *in vitro* (111, 112). *In vivo* chemotaxis mediated by these chemokines requires binding of chemokines to GAGs (111). Binding of chemokines to GAGs prevents diffusion of the ligand and probably has a protective function against proteolysis (113). Indeed, interference with GAG binding by using short chemokine-derived peptides, mutated chemokines without affinity for GPCRs, and enhanced-binding properties to GAGs, or chemokine-binding aptamers, which block chemokine–GAG interactions resulted in reduced leukocyte migration *in vivo* (114–116). In addition, *in vivo* heparin binding was shown to protect CCL11 (in)directly against proteolysis mediated by plasmin, cathepsin G, and elastin and positively affects the chemotactic activity of the chemokine (117). Evidence in favor of the hypothesis that GAG binding can serve as a protective factor regarding to proteolysis was also provided by the observation that binding to heparan sulfate or heparin oligosaccharides avoids CD26-mediated proteolysis of CXCL12 (118).

### Posttranslational Modification

In a number of immunological processes, the importance of posttranslational protein modifications has been evidenced, e.g., glycosylation patterns play a major role in the generation of blood groups, proteolytic cleavage in the activation of IL-1β and IL-18, and citrullination in the generation of autoantigens during rheumatoid arthritis (119–122). For chemokines, the biological availability is not only coordinated at the level of gene expression but also depends on interactions with GAGs and atypical receptors. Moreover, the exact chemokine function is regulated in detail by the presence or absence of synergistic or antagonistic effects of other chemokines, alternative splicing, and posttranslational modifications (13). Posttranslational modification of chemokines can result in an increase or a decrease of their biological activity and may affect their receptor specificity. The importance of this phenomenon has been underestimated for a long time. However, since chemokine isoforms have been identified *in vivo*, the impact of posttranslational modifications in the context of chemokine regulation receives higher recognition (19, 123, 124).

#### Proteolysis

Undoubtedly, the best known type of posttranslational modification on chemokines is proteolysis by specific enzymes (13, 19). Both the NH_2_-terminal and the COOH-terminal end of a chemokine can be subjected to proteolysis and internal cleavage by endopeptidases has also been described (124).

##### NH_2_-Terminal Proteolysis

Important enzymes regarding truncation of the NH_2_-terminal region of a chemokine are matrix metalloproteases (MMPs), CD26, cathepsins, elastase, and proteinase-3 (125). CD13, plasmin, and thrombin, which are present in plasma, can also mediate proteolytic chemokine modification. MMPs are produced by stromal cells and leukocytes and hold several CC as well as CXC chemokines as substrates. NH_2_-terminal proteolysis of CXCL8 by MMP-9 for example, but also by thrombin or plasmin, generates CXCL8(6–77), CXCL8(7–77), and CXCL8(9–77) (126–128). In case of CXCL8, the truncated chemokine forms act as more potent stimulators of MMP-9 secretion and are more powerful mediators of neutrophil chemotaxis than the intact chemokine. Moreover, binding of NH_2_-terminally truncated CXCL8 to the receptor initiates enhanced mobilization of intracellular calcium compared to the uncleaved chemokine. Ultimately, the proteolytic cleavage leads to a 10- to 30-fold increase in capacity to activate neutrophils *in vitro*. A recent study demonstrated a role for MMP-2 and MMP-9 at the blood–brain barrier, where these proteases promote chemokine-induced migration of leukocytes (129). For CXCL8, over 10 naturally occurring NH_2_-terminal forms have been described, most of these characterized by NH_2_-terminal truncation resulting in an increased biological activity (126, 130). In addition to ELR^+^CXC chemokines, such as CXCL8, several ELR^−^CXC chemokines are also MMP substrates. MMP-8-, MMP-9-, or MMP-12-mediated proteolysis of CXCL11, for example, generates a chemokine form with decreased chemotactic activity and an increased ability to bind heparin (131). For CXCL12, several NH_2_-terminal forms have been found in human plasma, which are all characterized by decreased activity or even an antagonistic effect with respect to the uncleaved chemokine (132). Concerning CC chemokines, MCPs are well known substrates for MMPs and, here, proteolysis results in inactivity (133). MMPs do not only cleave chemokines but also promote degradation of the extracellular matrix (134, 135). For some of these enzymes, increases in expression level are observed during conditions of inflammation and cancer (136–139). The role of cathepsins in posttranslational modification of chemokines has been studied to a lesser extent. In a recent study, it was shown that the cysteine cathepsins K, L, and S, by means of NH_2_-terminal proteolysis, provoke activation and inactivation of several ELR^+^- and ELR^−^CXC chemokines, respectively (140). In this study, GAGs turned out to partly reduce the enzymatic process. The role of the serine protease CD26 in NH_2_-terminal chemokine modification will be discussed in detail in a separate paragraph.

##### COOH-Terminal Proteolysis

More recently, the COOH-terminal domain of certain chemokines has been proven to be sensitive to proteolysis by specific enzymes. As mentioned, the domain is often important for interactions with GAGs (*vide supra*). For most chemokines, loss of a small number of amino acids in the COOH-terminal region does not have a biological effect, but more drastic modifications may negatively affect interactions between chemokines and GAGs. Again, members of the MMP family play an important role. For example, MMP-8 facilitates degradation of CXCL9 and CXCL10, and the same applies to MMP-9 (141). Worth mentioning is the effect of MMPs on CXCL11. Although NH_2_-terminal cleavage results in generation of a molecule with antagonistic properties and enhanced binding to heparin (*vide supra*), COOH-terminal proteolysis leads to a loss of antagonistic properties and heparin binding (131). These findings underscore the importance of the COOH-terminal region with respect to GAG binding and suggest that GAGs can modify the chemokine gradient in different directions. For CXCL12 – which is associated with lymphocyte migration and hematopoiesis – both termini can be subjected to proteolysis. Carboxypeptidase N-induced COOH-terminal proteolysis of this chemokine significantly reduces the biological activity (142). The membrane-associated chemokines CXCL16 and CX_3_CL1 can be subjected to proteolytic shedding as well. Furthermore, the intact membrane-associated form usually functions as an adhesion molecule and scavenger receptor, and can be converted into a soluble form by “a disintegrin and metalloprotease” (ADAM)-10 and ADAM-17 (143–145). The soluble forms act as chemoattractants for different types of immune cells, such as activated T cells (146). Increased concentrations of the soluble form usually reflect inflammation (124, 147, 148).

#### Citrullination, Nitration, and Glycosylation

The posttranslational modification, which affects the chemokine mass the least, is peptidylarginine deiminase (PAD)-catalyzed deimination of arginine to the uncharged amino acid citrulline (124). Since citrullination reduces the number of positive charges on proteins, it can change their 3D structure, and this modification has been reported to affect the interaction of proteins with lipids and GAGs (149–152). Furthermore, the sensitivity of a chemokine for proteolytic processing by serine proteases, which recognize positively charged residues can be affected (151). Protein citrullination and the presence of anti-citrullinated peptide antibodies have been linked to a number of specific pathological states including multiple sclerosis, rheumatoid arthritis, and psoriasis (121, 153–155). For chemokines, limited information is available on the importance of citrullination in a disease context. Increased concentrations of citrullinated CXCL5 have been found in serum and synovial fluid of patients with rheumatoid arthritis and correlated with disease activity (156). Compared to the unmodified molecule, citrullinated CXCL5 caused an enhanced monocyte but reduced neutrophil attraction in mice. In addition to citrullination of CXCL5, natural occurring citrullinated forms of CXCL8 and CXCL10 have been identified (151, 152). For CXCL8, citrullination was linked to protection of the chemokine against thrombin- and plasmin-mediated proteolytic processing, reduced affinity for GAGs, and reduced *in vivo* activity upon intraperitoneal injection in mice (151). Surprisingly, citrullination resulted in an increased capacity for CXCL8 to provoke mobilization of neutrophils into the blood circulation after intravenous injection in rabbits (157). Compared to the authentic chemokines, citrullinated forms of CXCL10 and CXCL11 were characterized by a diminished CXCR3 signaling capacity, impaired T cell chemotaxis, and reduced ability to bind to heparin (152). However, these citrullinated chemokines retained CXCR3 binding properties. Thus, although several natural citrullinated chemokines have been identified, their role in an *in vivo* context, either for the regulation of leukocyte homeostasis or during inflammation remains unknown. A particular problem with this modification is the difficulty to detect specific citrullinated proteins with high sensitivity. Classical immunoassays do not discriminate between the unmodified and citrullinated forms, and the minor mass difference (one mass unit) renders identification by mass spectrometry extremely difficult, in particular in patient-derived body fluids such as serum or synovial fluids.

*In vitro* nitration by peroxynitrite has been described for the chemokines CCL2, CCL5, and CXCL12 (158). Peroxynitrite is a highly reactive ion that is generated *in vivo* by the reaction of the free radicals superoxide and nitric oxide during sustained inflammation (159, 160). The unstable ion potently modifies several residues either directly (for methionine, tryptophan, and cysteine) or indirectly (for histidine, phenylalanine, and tyrosine) (161). The implications of nitration for chemokine function are divers. For CCL5, nitration was shown to significantly impair the potency of the chemokine to chemoattract eosinophils *in vitro* (162). Concerning CCL2, conflicting results have been reported. On the one hand, data were published, which claim that the ability of CCL2 to chemoattract antigen-specific CD8^+^ cells is reduced upon nitration, whereas the capacity of the chemokine to recruit CD14^+^ monocytes stays unaltered (163). On the other hand, nitration of CCL2 was linked to a reduction in monocyte chemotaxis (164). Recently, it was demonstrated for the first time that CXCL12 can be naturally nitrated on Tyr^7^ in an inflamed environment (165). Compared to unmodified CXCL12, the capacity of this novel and naturally occurring nitrated CXCL12 form to provoke intracellular calcium release was impaired. Nitration reduced the ability of CXCL12 to chemoattract monocytes and lymphocytes *in vitro* and, more importantly, nitrated CXCL12 was not longer able to recruit lymphocytes to the joints *in vivo*.

Last, some chemokines can be subjected to N- or O-glycosylation. The *in vivo* importance of this phenomenon is still to be elucidated. For example, the functional stability of CCL2 improves after O-glycosylation, but the *in vitro* chemotactic activity of the glycosylated molecule is not significantly altered (166, 167). In line with this observation, the *in vitro* chemotactic activities of CCL5 and CCL11 are also not influenced by glycosylation (168, 169). The limited availability of purified natural glycoforms of human chemokines and the difficulty to produce recombinant chemokines that contain specific sugar chains identical to the natural human sugars hampers profound investigation through *in vivo* studies.

## Dipeptidyl Peptidase IV/CD26

### Molecular Characteristics, Structure, and Signal Transduction

Dipeptidyl peptidase (DPP) IV was described for the first time by Hopsu-Havu and Glenner in 1966 as an active enzyme in livers of rats (170, 171). The serine protease is also named “adenosine complexing protein 2” or “cluster of differentiation 26” (CD26) and, nowadays, is considered to be the most important member of the DPP family (172). The family, strictly speaking, consists of four prolyl-specific peptidases, i.e., DPPIV/CD26, fibroblast-activating protein-α (FAP-α), DPP8, and DPP9, but, based on substrate specificity and structural homology, prolyl endopeptidase (PREP) or prolyl oligopeptidase (POP), DPPII, and prolyl carboxypeptidase (PRCP) are sometimes considered as family members as well. The DPP enzymes have been associated with a broad spectrum of physiological and pathophysiological processes of the immune system. In this review, we will specifically focus on CD26 in a chemokine context. The complex immunological roles of the activity and/or structural homologs of CD26 were recently reviewed by Wagner et al. (173).

The human CD26 gene contains 26 exons and is located on chromosome 2q.24.3. The gene spans a region of circa 70 kb (174). Flanking to the 5′ end, a sequence of 300 base pairs is located that consists for not less than 72% of cytosine and guanine residues, implying that the sequence holds potential-binding sites for growth factors such as the nuclear factor kappa-light-chain-enhancer of activated B cells (NF-κB) and hepatocyte nuclear transcription factor 1 (HNF-1). Absence of a TATA box and a high CG content, which characterize the CD26 gene, are typical features of a promotor region of a house keeping gene (175). The coding glycoprotein, as a monomer, has a size of 110 kDa and is multifunctional. CD26 exists both as a soluble molecule as well as in a membrane-bound form and functions as a serine protease, as a receptor, as an adhesion molecule for collagen and fibronectin, as a costimulatory signal for T lymphocytes, and is involved in apoptosis (176). Conditions of hypoxia promote CD26 expression and hypoxia-inducible protein-1α (HIP-1α) is a strong inducing factor for CD26 gene expression and protein production. Several cytokines including IFNs and IL-1β, retinoic acid, and HNF-1 can also stimulate activation of CD26 on fibroblasts, epithelial cells, endothelial cells, and leukocytes (177–180).

**Figure 3:**
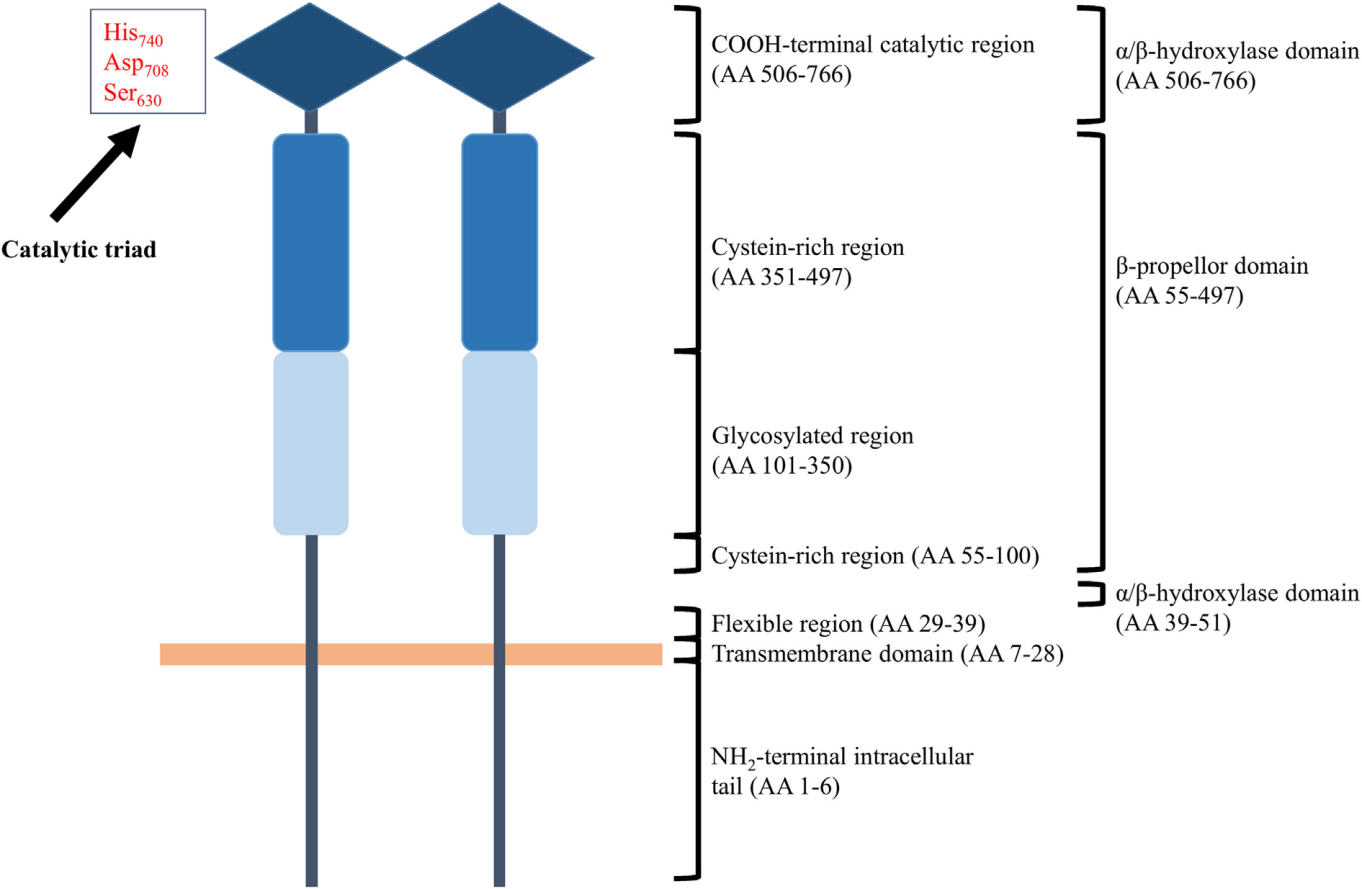
**Schematic structure of homodimeric CD26**. Each CD26 monomer consists of an intracellular NH_2_-terminal tail, a transmembrane region, a flexible part, a glycosylation-rich region, a cysteine-rich region, and a catalytic domain. Ser_630_, Asp_708_, and His_740_ are involved in the catalytic process and are generally referred to as “the catalytic triad.” Structurally, the two termini of a CD26 monomer contribute to the formation of a β-propeller structure. An α/β-hydroxylase domain is covalently bound to the β-propeller structure. These structural features imply that CD26’s catalytic pocket is situated in a locked hole.

Membrane-bound CD26 contains a transmembrane domain that is located 28 residues from the NH_2_-terminus and is a leukocyte surface marker (176, 181, 182). The protein shows catalytic proteolytic activity only as a dimer and can be found on the surface of T and B cells, NK cells, some types of macrophages, and hematopoietic stem and progenitor cells. In addition, fibroblasts, endothelial, acinar, and epithelial cells of different tissues like kidney and liver do also express CD26. Both termini of the protein contribute to the formation of a so called β-propeller domain (amino acids 55–497) (Figure 3). The β-propeller structure holds seven sheets and contains only hydrophobic bonds and salt bridges, implying that the region is extremely flexible. Furthermore, the protein contains an α/β hydroxylase domain (amino acid 39–51 and amino acid 506–766) that is covalently bound to the β-propeller domain. All together, these properties imply that the catalytic pocket is situated in a locked hole. The other side of the β-propeller domain faces the extracellular environment. It cannot be excluded that the flexibility of the β-propeller domain plays a role in facilitating the passage of substrates toward the catalytic pocket of CD26. However, only entrance of substrates through a side opening of the enzyme is supported by experimental data at the moment (183–185). In addition to functional homodimeric CD26, active heterodimers with FAPα have also been described (183).

CD26 contains multiple regions that can be subjected to N-glycosylation. Research, however, suggests that glycosylation of these sequences does not have implications for dimerization of the protein, binding to adenosine deaminase (ADA), or the catalytic activity (186). Residues in the catalytic pocket are highly conserved, and the presence of only point mutations is already sufficient to inhibit enzymatic functioning of CD26. The sequence at the height of the catalytic serine is G-W-S-Y-G implying that the protein meets the typical serine protease consensus motif, namely G-x-S-x-G (176, 182). Concerning its function as a serine protease, CD26 is highly specific: the protease cleaves NH_2_-terminal dipeptide sequences of proteins, only if a (hydroxy)proline or an alanine is present in the penultimate NH_2_-terminal position. The signal transduction cascade that is initiated by CD26 is not completely determined at the moment, but is, anyhow, associated with mobilization of intracellular calcium and partly involves the same substrates as those involved in T cell receptor-induced signal transduction, like stimulation of MAPK and PLCγ activity (182).

In addition to the membrane-bound protein, a soluble form of CD26 can be found in serum and in various body fluids, for example in seminal fluid (182). Soluble CD26 or sCD26 is – probably by means of proteolysis – derived from the membrane-bound form and has a stimulating effect on the proliferation of activated lymphocytes in peripheral blood. It has been demonstrated that MMP1, MMP2, MMP9, and MMP14 are able to facilitate release of soluble CD26 from adipocytes and vascular smooth muscle cells (187). The structures of sCD26 and membrane-bound CD26 are highly similar, the only difference being the absence of the cytoplasmic domains and the transmembrane domain for sCD26 (182). Like membrane-bound CD26, sCD26 is characterized by strong glycosylation. The degree of glycosylation increases with increasing age and some types of hypersialylation can inhibit CD26 activity (188). In addition to CD26, other circulating molecules with a similar activity exist. DPPII, for example, also cleaves the two NH_2_-terminal amino acids from substrates with a proline or an alanine residue in the penultimate NH_2_-terminal position (189, 190). Moreover, the activity of membrane-bound CD26 and sCD26 are possibly regulated by circulating attractin (189). Changes in sCD26 concentration and, in general, in CD26 activity, have been associated with a variety of pathophysiological conditions. Decreases in activity have been related to immune suppressive conditions and several tumor types. Reduced sCD26 concentrations, on the one hand, have been found in patients with systemic lupus erythromatosus, among others (191). Inflammatory and infectious conditions, liver diseases, and other types of tumors, on the other hand, are associated with increased CD26 activity and enhanced sCD26 levels. Increased CD26 expression as found in patients with T cell lymphoma or B cell leukemia, for example, is positively correlated to disease aggressiveness (192, 193).

### Functions

#### CD26 as Receptor, Costimulator, and Adhesion Molecule and Its Relation with Apoptosis

As a receptor, CD26 can interact with either receptor- or non-receptor molecules. Undoubtedly, the most reported binding partner of membrane-bound CD26 is ADA, an enzyme that catalyzes the irreversible conversion of adenosine and 2′-deoxyadenosine into, respectively, inosine and 2′-deoxyinosine and which plays a role in the development and functioning of lymphoid tissues (176, 194). Adenosine and 2-deoxyadenosine, in fact, have negative effects on the activation of both T and B cells. The direct association between ADA and CD26 ensures local degradation of adenosine and induces T cell proliferation. The ADA–CD26 complex is of importance in regulation of adhesion of T lymphocytes to epithelial cells. The interaction between the two enzymes, furthermore, functions as a costimulatory signal for T cell activation (195). Here, the signal transduction through CD26 is directly related to its expression level on T lymphocytes (196). Last, in the immunological synapse, interaction between the ADA–CD26 complex on T cells and ADA receptors on dendritic cells mediates increased release of pro-inflammatory cytokines such as IFN-γ, TNF-α, and IL-6 (197). Another enzyme that can be bound by CD26 is the zymogen plasminogen (198). Interaction with CD26 realizes its conversion to active plasmin. Plasmin promotes degradation of the extracellular matrix and, in this way, is involved in cell migration, tumor invasion, and metastasis.

The glycosylation domain of CD26 contains mannose 6-phosphate (M6P) residues that can interact with the M6P/insulin-like growth factor II-receptor (IGFIIR) (199). The complex formation results in internalization of CD26 and enables recycling of CD26 in hepatocytes and intestinal epithelial cells. Furthermore, this interaction with IGFIIR is required with respect to the function of CD26 as a costimulatory signal in T cell proliferation. The second receptor that can be subjected to interaction with CD26 is CXCR4 (200). This chemokine receptor selectively binds CXCL12 and binding of the latter to the CXCR4–CD26 complex provokes internalization of both receptors. In this way, the CXCR4–CD26 complex probably regulates the local activity of CXCL12, and CD26 seems to exert a direct influence on the antiviral activity of CXCL12, its hematopoietic effects, and its functionality as a chemoattractant (200–202).

A high content of Gly-Pro sequences makes collagen a potential CD26 interaction partner. In 1988, it became clear that CD26, as expected, facilitates the spreading of hepatocytes *in vitro* and enhances binding of the cells to natural collagen *in vitro* (203). The domain that is responsible for these interactions is situated in the cysteine-rich region of the enzyme. By means of interacting with the extracellular matrix proteins collagen and fibronectin, CD26 is involved in processes of cell adhesion and migration (204). Disruption of the adhesion-promoting functions of CD26 has been associated with various pathophysiological conditions. For example, cultured hepatoma cells show decreased membrane expression of CD26 protein, increased sCD26 levels, and loss of adhesion (205). Furthermore, the protein functions as a collagen receptor in the activation of CD4^+^ lymphocytes (206).

The influence of the multifunctional CD26 protein on apoptosis probably depends on the context and the particular cell type involved (176). In several liver cell lines, for example, CD26 is physically associated with a tyrosine kinase and possesses apoptosis-promoting properties (207). On the other hand, parental Jurkat T cells that are CD26 negative or that are transfected with mutant CD26, show increased expression of the cell death-associated Fas receptor CD95 and, compared to the CD26 positive parenteral Jurkat T cells, are more prone to be subjected to apoptosis (208).

#### Enzymatic Activity of CD26

The serine protease CD26 typically shows a strong selectivity for short chain peptides whose penultimate position in the NH_2_-terminal amino acid sequence is occupied by a (hydroxy)proline or an alanine residue (176, 209). Substrate binding and catalysis are realized by the catalytic pocket, with the rate of the proteolytic process being inversely related to the chain length of the substrate. The substrate selectivity of the enzyme is reflected by the k_cat_/K_M_ ratio. Here, k_cat_ and K_M_, respectively, represent the catalytic- and the Michaëlis–Menten constant, and the mutual ratio reflects the selectivity constant. The latter can be used as a measure for the half-life of a substrate at a fixed enzyme concentration. Notation of positions of residues relatively to the scissile bond is in accordance with the classification system of Schechter and Berger that dates from 1968 (210). Amino acids in the direction of the NH_2_-terminus are represented by P_1_, P_2_, etc. and residues in the direction of the COOH-terminus are referred to as ${\text{P}}_{\text{1}}\prime ,{\text{ P}}_{2}\prime$, etc. CD26 shows a strong selectivity for substrates with a (hydroxy)proline or an alanine in the P_1_ position and is stereo specific: the scissile bond and the bond between P_1_ and P_2_ have to be in trans configuration (211).

### CD26 and Chemokines

The relationship between CD26 and the immune system extends beyond expression of the membrane-bound enzyme on immune-related cells such as B and T lymphocytes. Indeed, many chemokines hold a proline or an alanine residue in the penultimate position of their NH_2_-terminal sequence. Presence of this motif, which is strongly preferred by CD26, suggests that the chemotactic proteins are sensitive to proteolysis by the enzyme. Based on *in vitro* as well as *in vivo* research, several chemokines have been identified as CD26 substrates (Table 1) (13, 19, 123, 124, 212–214). As mentioned before, the NH_2_-terminal domain determines the capacity of chemokines to activate GPCRs. Therefore, it is not surprising that limited proteolysis within this region can affect the interaction between chemokines and their receptors (Figure 1). The effect of enzymatic cleavage turned out to be highly complex and, depending on the chemokine and the type of truncation, can result in either an increase or a decrease in the biological activity, can alter the chemokine’s receptor preference, or generate receptor antagonists. The fact that chemokines are involved in a broad range of crucial cellular processes in pathological as well as physiological conditions underlines the potential implications of CD26-mediated proteolysis in the context of chemokines.

**Table 1:** **Overview of the chemokines that have been identified as CD26 substrates**.

Chemokine	Amino acids in the truncated form	Type of research	Original source of cleaved chemokine	Biological effect	Reference
**(I) CC chemokines**					
CCL3L1	3–70^a^	*In vivo*, indirect	Mononuclear cells of peripheral blood	Increased on CCR1 and CCR5; decreased on CCR3	(19, 54, 55, 189)
CCL4	3–69^a^	*In vivo*, indirect	Activated peripheral lymphocytes	Increased on CCR1 and CCR2, unchanged on CCR5	(19, 189, 215, 216)
CCL5	3–68^a^	*In vitro*, indirect	Cytokine-stimulated fibroblasts, sarcoma cells, and leukocytes	Decreased on CCR1 and CCR3, increased on CCR5	(19, 56, 189, 217–220)
CCL11	3–74^a^	*In vitro*; *in vivo* in rats	Dermal fibroblasts	Decreased activity on CCR3, inhibitor of intact CCL11	(19, 169, 189, 221–223)
CCL22	3–69^a^	*In vitro*	Transformed CD8^+^ T lymphocytes	Decreased on CCR4, monocyte chemotaxis unaffected	(19, 189, 224, 225)
	5–69				
**(II) CXC chemokines**					
CXCL2	3–73	*In vitro*	–	Unknown	(19)
CXCL6	3–77^a^	*In vitro*	Cytokine-stimulated MG-63 osteosarcoma cells	Preserved effect on neutrophils	(19, 189, 226)
CXCL9	3–103	*In vitro*	–	Inactivity, decreased effect on T cells	(19, 189, 227)
CXCL10	3–77^a^	*In vivo*, indirect	Cytokine-stimulated fibroblasts and MG-63 osteosarcoma cells	Inactivity, decreased effect on T cells, and CXCR3 inhibition	(19, 180, 189, 226–228)
		*In vivo* in mice			
CXCL11	3–73^a^	*In vivo*	IFN-γ-stimulated keratinocytes	Inactivity, decreased effect on T cells, and CXCR3 inhibition	(19, 189, 227, 229–231)
CXCL12α	3–68	*In vivo*, indirect	Blood plasma (human, mouse, and rhesus monkey) in physiological conditions	Inactivity, decreased effect on lymphocytes, CXCR4 inhibition	(19, 133, 189, 232–235)
CXCL12β	3–72^a^	*In vivo*, indirect	Can be found in blood plasma from human, mouse, and rhesus monkey in physiological conditions	Unknown	(19, 133, 189, 234)

*Several CC and CXC chemokines were identified as potential CD26 substrates. For many of these chemokines, the NH_2_-terminal truncated form was successfully isolated from natural resources (^a^) and the effect of CD26-mediated proteolysis on receptor selectivity and biological activity was examined. “Indirect” refers to a lack of *in vivo* or *in vitro* studies with inhibitors. Studies that are mentioned following the heading “type of research” are human studies unless indicated differently*.

#### CC Chemokines

Based on *in vitro* and *in vivo* experiments, several CC chemokines were identified as CD26 substrates. Although the three human MCPs have an NH_2_-terminal penultimate Pro, the presence of a pyroglutamic acid – which results from cyclization of an NH_2_-terminal glutaminyl precursor molecule – in all three molecules (CCL2, CCL7, and CCL8) protects these natural human chemokines from cleavage by CD26 (236). However, when MCPs are produced in bacteria as recombinant proteins, they have an NH_2_-terminal glutamine instead of a pyroglutamic acid and become artificial CD26 substrates. Upon storage at neutral or slightly basic pH conditions, the glutamine in the recombinant chemokines will spontaneously convert into a pyroglutamic acid. Interestingly, for human and murine CCL2, the enzyme responsible for NH_2_-terminal cyclization into pyroglutamic acid was demonstrated to be the isoenzyme of glutaminyl cyclase (237). Moreover, inhibition of this enzyme by using small orally available molecules reduced the monocyte filtration induced by murine CCL2 in an *in vivo* atherosclerosis model. These observations support the idea that interfering with chemokine cleavage is of potential clinical value.

Most of the CD26-truncated human CC chemokine forms were successfully isolated from natural resources [(Table 1) (I)]. An individual CC chemokine usually activates multiple receptors and NH_2_-terminal proteolysis, in most cases, implies a change in receptor preference. Truncated CC chemokines might show increased affinity for a specific receptor or, in contrast, a reduced affinity or missing activity, resulting in receptor inhibition. Indeed, in this way, proteolysis induced by CD26 often interferes with the chemotactic properties of these chemokines.

##### CCL3L1 and CCL3

A clear pro-inflammatory effect of CD26-mediated proteolysis was demonstrated for the first time for the chemokine CCL3L1. Indeed, the truncated form CCL3L1(3–70) shows increased affinity for CCR1 (54). Consequently, the pro-inflammatory properties of the chemokine, together with the chemotactic activity for monocytes, are enhanced. In addition to being an outstanding CCR1 agonist, the affinity of the truncated chemokine for CCR5 remains very strong and, as a consequence, the modified CCL3L1 form owns potent anti-HIV-1 activity. Noteworthy, it was recently shown that CD26-mediated proteolytic processing of the murine CCL3, which is more related to human CCL3L1 than to human CCL3 due to a proline residue in the penultimate NH_2_-terminal position, results in loss of myelosuppressive activity *in vitro* (202). Moreover, the truncated form acts as an inhibitor with respect to the myelosuppressive activity of the corresponding full length chemokine. These data were confirmed in mice. However, as it was demonstrated that the myelosuppressive activity of CCL3 was not mediated through CCR1 or CCR5, the two known receptors for CCL3, it remains to be elucidated *via* which receptor CCL3 manifests this myelosuppression (202, 238).

##### CCL4

The β chemokine CCL4, together with CCL3, is released by T lymphocytes and monocytes (216). The intact chemokine shows a selective CCR5 affinity and holds, by means of receptor occupation and receptor downregulation, anti-HIV-1 activity. CCR5 is expressed by macrophages and lymphocytes, implying that CCL4 directs migration of these CCR5 positive cells toward lymph nodes or other tissues. CCL4(3–69) is secreted by activated T lymphocytes, shows preserved effects on CCR5 and, moreover, acquires affinity for CCR1 and CCR2 (215, 216, 239). The change in receptor specificity that characterizes CCL4(3–69) suggests that the truncated CCL4 form plays a role in chemotaxis and migration of CCR1 and CCR2 positive cells, such as monocytes, immature dendritic cells, and lymphocytes. It has been demonstrated that CCL4(3–69), in contrast to intact CCL4, no longer enhances proliferation of single cytokine-sensitive hematopoietic progenitor cells *in vitro* (202). Moreover, the truncated CCL4 form inhibits the enhancing activity of the intact chemokine.

##### CCL5

In the context of CD26-mediated proteolytic processing of CC chemokines, CCL5 was the first chemokine that was examined in detail. The chemokine promotes recruitment of, among others, monocytes, eosinophils, basophils, and NK cells *via* activation of three different receptors. The NH_2_-terminally truncated form CCL5(3–68) was successfully isolated from natural sources including conditioned media of fibroblasts, sarcoma cells, and leukocytes and shows a deviating receptor selectivity compared to the intact chemokine (56, 217). CCL5(3–68) is a more powerful activator of CCR5, but has lost affinity for CCR1 and CCR3 (56, 218). The modified chemokine is still chemotactic for lymphocytes, but no longer owns the capacity to chemoattract monocytes and eosinophils, and, moreover, acts as an inhibitor with respect to chemotaxis induced by CCL5(1–68), CCL3, CCL4, and CCL7. In addition to a function as a chemokine receptor, CCR5 acts as an important co-receptor for the human immunodeficiency virus (HIV). Consequently, CCL5 competes with the virus for binding to the receptor. Since the affinity of CD26-truncated CCL5 for the CCR5 receptor is remarkably higher, CCL5(3–68) has been found to be an extremely potent HIV-1 inhibitor (219, 220).

##### CCL11

As suggested by its original name – being eotaxin – CCL11 is an important chemoattractant for eosinophilic granulocytes. These cells facilitate defense against multicellular parasites and are involved in the mechanisms that underlie allergic asthma. The truncated isoform CCL11(3–74) has lost affinity for CCR3 and, consequently, no longer holds chemotactic activity for eosinophils (222). In an *in vivo* study, it was demonstrated that CD26-deficient rats and wild-type animals that were treated with a CD26 inhibitor, characteristically show enhanced mobilization of eosinophils compared to wild-type animals (221). These findings imply a potential role for CD26 as regulator of eosinophil recruitment.

##### CCL22

CCL22 is secreted by macrophages and dendritic cells. The chemokine interacts with CCR4 and facilitates chemotaxis of activated T lymphocytes, monocytes, NK cells, and dendritic cells. The capacity to activate CCR4 decreases following proteolysis by CD26 and, consequently, also the capacity of CCL22 to direct lymphocyte chemotaxis is lost (240). CCL22(3–69) has preserved monocyte chemotactic properties and, moreover, shows increased anti-HIV-1 activity compared to the intact chemokine form (224, 225, 240). CCR4, at the moment, is the only receptor for CCL22 that has been identified, but is currently not described to be a co-receptor for HIV-1. For this reason, the increased antiviral capacity of CCL22 is highly remarkable and suggests existence of an additional CCL22 receptor (225). *In vitro*, CD26 has been shown to facilitate the unexpected proteolytic conversion of CCL22(3–69) into CCL22(5–69) (240). This is remarkable given that the protease, which generally shows strong selectivity for dipeptides with a (hydroxy)proline or an alanine in the penultimate NH_2_-terminal position, in this case, cleaves behind a Tyr-Gly dipeptide.

#### CXC Chemokines

##### ELR^+^CXC Chemokines

ELR^+^CXC chemokines activate CXCR1 and/or CXCR2 and facilitate chemotaxis of neutrophils. In general, they are angiogenic. NH_2_-terminal proteolysis has direct consequences for the receptor affinity and biological activity of almost all ELR^+^CXC chemokines including CXCL8 (*vide supra*). Most ELR^+^CXC chemokines, with the exception of CXCL2 and CXCL6, are no CD26 substrates because they do not possess a penultimate NH_2_-terminal proline or alanine. Knowledge concerning the role of CD26 in posttranslational modification of CXCL2 and CXCL6 is limited. CD26 cleaves CXCL2 and also CXCL6 *in vitro* (241). Natural human CXCL6(3–77), isolated from stimulated osteosarcoma cells, does not show a significantly altered *in vitro* chemotactic activity compared to the intact form on neutrophils (219, 226). Noteworthy, the mouse chemokine CXCL1 – in contrast to human CXCL1 – contains a proline residue in the penultimate position, implying that the chemokine is also a potential substrate for CD26 (242).

##### ELR^−^CXC Chemokines

The three interferon-induced ELR^−^CXC chemokines, i.e., CXCL9, CXCL10, and CXCL11, interact with CXCR3 and induce chemotaxis of activated T lymphocytes and NK cells (243). Most ELR^−^CXC chemokines, with exception of CXCL12 and CXCL16, are angiostatic. NH_2_-terminal truncated forms of the CXCR3 agonists CXCL9, CXCL10, and CXCL11 show preserved antiangiogenic properties but their capacity to trigger CXCR3-mediated signal transduction is limited (227). As a consequence, the capacity of the truncated chemokine forms to provoke lymphocyte chemotaxis is significantly reduced. Recently, it was shown that CXCL9, following proteolytic processing by CD26, no longer acts as a myelosuppressive protein *in vitro* and *in vivo* (202). Instead, the truncated form counteracts the myelosuppressive effects of the corresponding intact chemokine.

Among the CXCR3 agonists, CD26-mediated proteolysis of CXCL11, in particular, turned out to be highly efficient with half-lives of the chemokines in the range of minutes in the presence of physiological concentrations of sCD26. The resulting CXCL11(3–73), as well as the CXCL10 form CXCL10(3–77), were isolated from natural sources (180, 229). Moreover, proteolytic processing of CXCL10 by CD26 was demonstrated in mice (228). Higher concentrations of intact CXCL10 were found in mice that were treated with the CD26 inhibitor sitagliptin compared to animals that did not receive the inhibitor. In contrast to sitagliptin-treated mice, untreated animals showed reduced infiltration of leukocytes into tumor tissue and a significantly decreased natural antitumor immunity. This is in line with former data that indicate that the truncated form CXCL10(3–77) is biologically inactive (227). Recently, based on two prospective clinical trials, it was confirmed that, in human, CD26 cleaves CXCL10 *in vivo* (244). This study for the first time provided direct *in vivo* evidence in favor of inhibition of CD26 in human to preserve intact CXCL10 by means of protecting the chemokine against proteolytic processing by CD26, which generates biologically inactive CXCL10(3–77). Noteworthy, biologically inactive CXCL10(3–77) was previously found in plasma from patients that suffer from a chronic viral hepatitis C infection (245).

CXCL12α acts as a strong chemoattractant for lymphocytes and CD34^+^ hematopoietic progenitor cells (246). Through interaction with CXCR4, together with CCR5 the most important co-receptor for HIV-1, this chemokine exerts potent antiviral activity. NH_2_-terminal proteolysis of this CD26 substrate negatively affects its capacity to bind CXCR4, which is reflected in a significantly reduced chemotactic- and antiviral activity of CXCL12α(3–68) compared to the intact chemokine (232). For this reason, the relationship between CD26 and HIV-1 seems to be dual: while CCL5(3–68) is characterized by increased anti-HIV-1 activity (*vide supra*) through enhanced interaction with CCR5, CXCL12α(3–68) is a less potent inhibitor of the virus compared to intact CXCL12α due to reduced affinity for CXCR4. Directly in line with the former results that suggest that CXCL12α(3–68) is biologically inactive, it was shown that CXCL12-mediated homing of hematopoietic progenitor cells *in vivo* is enhanced in *Cd26*^−/−^ mice and wild-type animals that received the CD26 inhibitor diprotin A, compared to untreated wild-type animals (247). The CD26 inhibitor protects CXCL12 from inactivation, resulting in enhanced cell migration, which is reflected in increased efficiency of cell homing after bone marrow transplantation. Noteworthy, an earlier study revealed that CD26 not only negatively affects the capacity of CXCL12 to mediate progenitor cell survival but also acts as a more general negative regulator of colony-stimulating factor activity and stress hematopoiesis through cleavage of growth factors and cytokines including granulocyte-macrophage colony-stimulating factor, granulocyte colony-stimulating factor, erythropoietin, and IL-3 (248, 249). In line with the observations from animal studies, it was found that inhibition of CD26 enhances clinical cord blood transplantation in humans with hematological malignancies (250, 251). Furthermore, the CD26–CXCR4 axis seems to play an important role in oncology. Cancer cells derived from patients with Sézary syndrome, for example, typically show absence of membrane-bound CD26 and increased expression of CXCR4 and CXCL12 (252). As a consequence, the chemokine is no longer inactivated, and the neoplastic cells show outstanding migratory properties. The latter process can be strongly reduced by administration of sCD26.

### Inhibition of CD26

#### Relevance and Implications

It becomes more and more evident that the multifunctional serine protease CD26 is associated with a variety of pathophysiological and physiological processes. In addition to chemokines, a broad range of regulatory peptides, mainly those involved in glucose metabolism, have been identified as substrates for this peptidase (189). For this reason, a range of different inhibitors are already used *in vitro* and *in vivo* and some inhibitors have been approved for treatment of diabetes patients. The principle of inhibition is always based on interaction of these molecules with the catalytic region of CD26, but further characteristics of specific inhibitors are highly diverse. For example, the inhibitor–enzyme interaction can be reversible or irreversible, an inhibitor can be a product analog as well as a substrate analog, inhibition can be competitive or non-competitive, etc.

In Europe and the United States, the reversible and competitive inhibitors sitagliptin, saxagliptin, vildagliptin, alogliptin, and linagliptin are already available on the market for therapeutic use (209, 253, 254). With regard to older studies that are based on the use of CD26 inhibitors, it is recommended to interpret the evidence of the results with some caution. A number of the CD26 inhibitors that have been used in these studies turned out to inhibit DPP8 and DPP9 as well, suggesting that the observed effects may not be attributed to inhibition of CD26 only (255).

#### CD26 Inhibitors

For some regulators of glucose homeostasis and insulin secretion, it has been confirmed that these molecules are sensitive to NH_2_-terminal proteolysis by CD26. Glucose-dependent insulinotrophic polypeptide (GIP) and gastrointestinal hormone glucagon-like peptide 1 (GLP-1), among others, are proteolytically processed by CD26 *in vitro* as well as *in vivo* (256). GLP-1 is postprandially secreted by L cells in the small intestine and colon and stimulates both *in vitro* and *in vivo* insulin secretion. Cleavage of the NH_2_-terminal region generates biologically inactive des(His-Ala)-GLP-1. In addition, CD26-mediated truncation of GIP results in inactivity as well. Ultimately, generation of the two antagonists leads to the development of insulin resistance. Accordingly, the therapeutic benefit for diabetic patients that are treated with CD26 inhibitors, is not surprising (257). The role of CD26 in regulation of neuropeptide activity falls beyond the scope of this review; however, it is worth mentioning that also CD26-mediated processing of neuropeptide Y, for example, affects its receptor preference (258, 259). Thus, it became clear that CD26-provoked NH_2_-terminal truncation affects the function of multiple peptide families with physiological consequences on glucose metabolism, immunological, and neurological responses.

In 2006, the first in class CD26 inhibitor sitagliptin became available on the market for use as an oral anti-hyperglycemicum. The drug is a selective and competitive inhibitor that binds CD26 non-covalently and in a reversible manner (260). Inhibition of the serine protease, mediated by sitagliptin, rests on the principle of structure–activity relationships.

Here, interaction with sitagliptin negatively affects the relationship between the structure and the biological activity of CD26. More specifically, a hydrogen bond is formed between the drug and amino acid Tyr_547_ of CD26. As a result, the residue is no longer able to facilitate stabilization of the active pocket of the enzyme.

Use of CD26 inhibitors, such as sitagliptin, is considered as a recent and revolutionary approach in the treatment of type 2 diabetes. The underlying mechanism rests on prevention of proteolytic cleavage of GIP and GLP-1, by means of which insulin secretion and β cell proliferation are facilitated on the one hand and, on the other hand, glucagon secretion and apoptosis are inhibited (261). As a drug, a very favorable safety profile characterizes sitagliptin. The inhibitor, in general, is well tolerated and the adverse effects that have been described are only mild or moderate and of low incidence (262). Sitagliptin is excreted unchanged for over 80% by means of renal excretion and is subjected to metabolism to a limited extent only. The latter process is realized by the cytochrome P450 enzymes CYP3A4 and CYP2C6 and the metabolites that are formed are completely inactive (263, 264).

In addition to sitagliptin, the CD26 inhibitors saxagliptin, vildagliptin, alogliptin, and linagliptin are approved for therapeutic purposes in Europe and the United States (254). However, sitagliptin and alogliptin are considered to be the only inhibitors that selectively inhibit CD26 and no other related peptidases. Excluding linagliptin, which is almost completely bound to proteins when in circulation, inhibitors generally show only limited and reversible protein binding (265, 266). As a consequence, linagliptin is primarily hepatically cleared. This is in direct contrast with other inhibitors that are primarily renally excreted. Among the five approved inhibitors, vildagliptin is the only one that needs to be dosed twice a day instead of once daily. Moreover, vildagliptin is associated with an increase in hepatic enzymes and, consequently, is not recommended in patients with mild to moderate hepatic insufficiency.

The wide range of potential CD26 substrates, together with the fact that CD26 has been associated with a variety of pathological processes, support the idea that the serine protease is an interesting drug target and suggest a broader scope of application for use of CD26 inhibitors [reviewed in detail in Ref. (267)]. Concerning chemokines, it was recently demonstrated that sitagliptin-treated mice, following intravenous stimulation with CpG, show increased concentrations of intact CXCL10 and increased antitumor activity compared to animals that did not receive the CD26 inhibitor (228). Moreover, it had been shown that sitagliptin *in vivo* blocks CD26-mediated truncation of CXCL10 in human (244).

## Concluding Remarks

Since the identification of chemokine isoforms both *in vitro* and *in vivo*, the role of posttranslational modifications of chemokines in the regulation of the biological activity of these chemotactic proteins can no longer be neglected. Over the years, it became clear that many chemokines can be subjected to proteolytic processing by the serine protease DPP IV or CD26. The enzyme cleaves the two most NH_2_-terminal residues from substrates with a proline or alanine in the penultimate position. The NH_2_-terminal domain of chemokines is crucial for their interaction with chemokine receptors and, consequently, CD26-mediated proteolysis can have significant implications for the receptor specificity and the chemotactic activity of chemokines. Indeed, chemokines are crucial for correct leukocyte migration in pathophysiological as well as homeostatic conditions and, therefore, they are essential for proper functioning of the immune system. Consistently, proteolytic processing by CD26 potentially has major effects on the organism’s well-being. Results from *in vivo* studies in mice with CD26 inhibitors provide evidence for this hypothesis. More research concerning the role of CD26-mediated chemokine processing *in vivo* in human will be necessary; however, the existing data support the idea that the enzyme plays a significant role in chemokine modification and that CD26-inhibitors are of potential therapeutic value in treatment of a variety of pathological conditions. In-depth epidemiological analysis of the progression of inflammatory diseases in diabetes patients who have been treated with CD26 inhibitors during the last decade will be an important step to unravel the role of CD26 in chemokine-mediated inflammatory processes.

## Author Contributions

MM wrote the initial version of the manuscript with assistance of AM and PP. The manuscript was further modified by JVD, AM, and PP and approved by all the authors.

## Conflict of Interest Statement

The authors declare that the research was conducted in the absence of any commercial or financial relationships that could be construed as a potential conflict of interest.

## Abbreviations

ADA, adenosine deaminase; ACKR, atypical chemokine receptor; CCR, CC chemokine receptor; CD26, cluster of differentiation 26; CX_3_CR, CX_3_C chemokine receptor; CXCR, CXC chemokine receptor; DAG, diacylglycerol; DPP, dipeptidyl peptidase; ER, endoplasmic reticulum; ERK, extracellular signal-regulated kinase; FAP-α, fibroblast-activating protein-α; GAG, glycosaminoglycan; GIP, glucose-dependent insulinotrophic polypeptide; GLP-1, gastrointestinal hormone glucagon-like peptide 1; GPCR, G protein-coupled receptor; GRKs, G protein-coupled receptor kinases; GTPase, guanosine triphosphatase; HIP-1α, hypoxia-inducible protein-1α; HIV, human immunodeficiency virus; HNF-1, hepatocyte nuclear transcription factor 1; IFN, interferon; IL, interleukin; IGFIIR, insulin-like growth factor II-receptor; IP_3_, inositol 1,4,5-trisphosphate; M6P, mannose 6-phosphate; MAPK, mitogen-activated protein kinase; MCP, monocyte chemotactic protein; MIP, macrophage inflammatory protein; NF-κB, nuclear factor kappa-light-chain-enhancer of activated B cells; NK, natural killer; PAD, peptidylarginine deiminase; PAMP, pathogen-associated molecular patterns; PI3K, phosphatidylinositol 3-kinase; PIP_2_, phosphatidylinositol 4,5-bisphosphate; PIP_3_, phosphatidylinositol (3,4,5)-trisphosphate; PKB, protein kinase B; PKC, protein kinase C; PLC, phospholipase C; POP, prolyl oligopeptidase; PRCP, prolyl carboxypeptidase; PREP, prolyl endopeptidase; TNF, tumor necrosis factor; XCR, C chemokine receptor.
